# Prediction of fluid responsiveness in septic shock patients: comparing automated pulse pressure variation by IntelliVue MP monitor and stroke volume variation by FloTrac™/Vigileo™

**DOI:** 10.1186/cc9473

**Published:** 2011-03-11

**Authors:** B Khwannimit, R Bhurayanontachai

**Affiliations:** 1 Songklanagarind Hospital, Songkhla, Thailand; 2 Division of Critical Care Medicine, Hat Yai, Thailand

## Introduction

The aim of this study was to assess and compare the ability of the automatically and continuously measured pulse pressure variation (PPV) obtained by an IntelliVue MP monitor and stroke volume variation (SVV) measured by FloTrac™/Vigileo™ to predict fluid responsiveness in septic shock patients.

## Methods

We conducted a prospective study on 42 mechanically ventilated septic shock patients. SVV, PPV and other hemodynamic data were recorded before and after fluid administration with 500 ml of 6% tetrastarch. Responders were defined as patients with an increase in cardiac index ≥15% after fluid loading.

## Results

**Figure 1:**
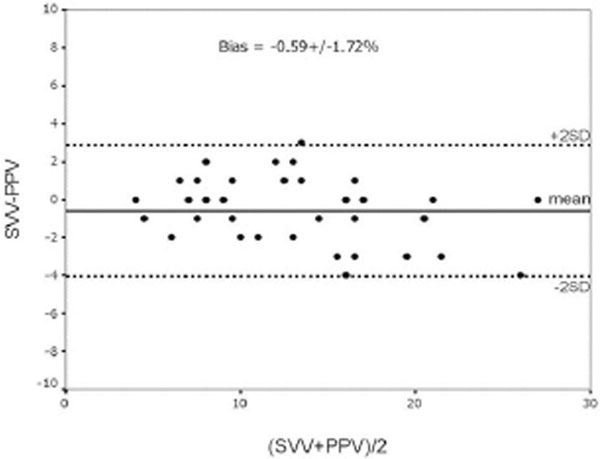
**Bland-Altman analysis for the agreement between SVV and PPV**.

The agreement (mean bias ± SD) between PPV and SVV was -0.59 ± 1.72% (Figure 1). The baseline PPV correlated with the baseline SVV (*r *= 0.96, *P *< 0.001). Twenty-seven (64.3%) patients were classified as fluid responders. PPV and SVV were significantly higher in responders than in nonresponders (16.2 ± 4.9 vs. 7.1 ± 2% and 15.3 ± 4.3 vs. 6.9 ± 1.9%, respectively, *P *< 0.001 for both). There was no difference between the area under the receiver operating characteristic curves of PPV (0.983) and SVV (0.99). The optimal threshold values to predicting fluid responsiveness were 10% for PPV (sensitivity 92.6%, specificity 86.7%) and 10% for SVV (sensitivity 92.6%, specificity 100%).

## Conclusions

The automated PPV, obtained by the Intellivue MP monitor, and the SVV, obtained by FloTrac™/Vigileo™, showed comparable performance in terms of predicting fluid responsiveness in mechanically ventilated patients with septic shock.
